# Enhanced Information Exclusion Relations

**DOI:** 10.1038/srep30440

**Published:** 2016-07-27

**Authors:** Yunlong Xiao, Naihuan Jing, Xianqing Li-Jost

**Affiliations:** 1 School of Mathematics, South China University of Technology, Guangzhou 510640, China; 2 Max Planck Institute for Mathematics in the Sciences, Leipzig 04103, Germany; 3 Department of Mathematics, North Carolina State University, Raleigh, NC 27695, USA

## Abstract

In Hall’s reformulation of the uncertainty principle, the entropic uncertainty relation occupies a core position and provides the first nontrivial bound for the information exclusion principle. Based upon recent developments on the uncertainty relation, we present new bounds for the information exclusion relation using majorization theory and combinatoric techniques, which reveal further characteristic properties of the overlap matrix between the measurements.

Mutual information is a measure of correlations and plays a central role in communication theory^1^,^2^,^3^. While the entropy describes uncertainties of measurements^4^,^5^,^6^,^7^,^8^, mutual information quantifies bits of gained information. Furthermore, information is a more natural quantifier than entropy except in applications like transmission over quantum channels^9^. The sum of information corresponding to measurements of position and momentum is bounded by the quantity log2Δ*X*Δ*P*_*X*_/*ħ*; for a quantum system with uncertainties for complementary observables Δ*X* and Δ*P*_*X*_, and this is equivalent to one form of the Heisenberg uncertainty principle^10^. Both the uncertainty relation and information exclusion relation^11^,^12^,^13^ have been used to study the complementarity of obervables such as position and momentum. The standard deviation has also been employed to quantify uncertainties, and it has been recognized later that the entropy seems more suitable in studying certain aspects of uncertainties.

As one of the well-known entropic uncertainty relations, Maassen and Uffink’s formulation^8^ states that


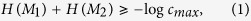


where 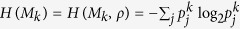 with 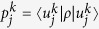 (*k* = 1, 2; *j* = 1, 2, …, *d*) for a given density matrix *ρ* of dimension *d*, and 
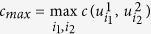
, and 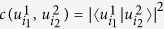 for two orthonormal bases 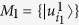 and 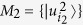 of *d*-dimensional Hilbert space 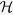.

Hall^11^ generalized Eq. (1) to give the first bound of the *Information Exclusion Relation* on accessible information about a quantum system represented by an ensemble of states. Let *M*_1_ and *M*_2_ be as above on system *A*, and let *B* be another classical register (which may be related to *A*), then


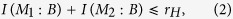


where 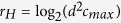 and *I*(*M*_*i*_ : *B*) = *H*(*M*_*i*_) − *H*(*M*_*i*_|*B*) is the *mutual information*^14^ corresponding to the measurement *M*_*i*_ on system *A*. Here *H* (*M*_*i*_|*B*) is the conditional entropy relative to the subsystem *B*. Moreover, if system *B* is quantum memory, then 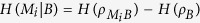 with 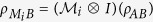, while 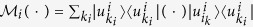. Eq. (2) depicts that it is impossible to probe the register *B* to reach complete information about observables *M*_1_ and *M*_2_ if the maximal overlap *c*_*max*_ between measurements is small. Unlike the entropic uncertainty relations, the bound *r*_*H*_ is far from being tight. Grudka *et al*.^15^ conjectured a stronger information exclusion relation based on numerical evidence (proved analytically only in some special cases)


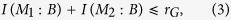


where 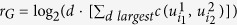. As the sum runs over the *d* largest 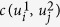, we get 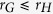, so Eq. (3) is an improvement of Eq. (3). Recently Coles and Piani^16^ obtained a new information exclusion relation stronger than Eq. (3) and can also be strengthened to the case of quantum memory^17^


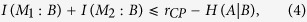


where *r*_*CP*_ = min {*r*_*CP*_(*M*_1_, *M*_2_), *r*_*CP*_(*M*_2_, *M*_1_)}, 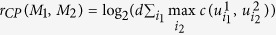, and *H*(*A*|*B*) = *H*(*ρ*_*AB*_) − *H*(*ρ*_*B*_) is the conditional von Neumann entropy with 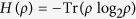 the von Neumann entropy, while *ρ*_*B*_ represents the reduced state of the quantum state *ρ*_*AB*_ on subsystem *B*. It is clear that 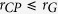.

As pointed out in ref. 11, the general information exclusion principle should have the form


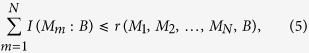


for observables *M*_1_, *M*_2_, …, *M*_*N*_, where *r*(*M*_1_, *M*_2_, …, *M*_*N*_, *B*) is a nontrivial quantum bound. Such a quantum bound is recently given by Zhang *et al*.^18^ for the information exclusion principle of multi-measurements in the presence of the quantum memory. However, almost all available bounds are not tight even for the case of two observables.

Our goal in this paper is to give a general approach for the information exclusion principle using new bounds for two and more observables of quantum systems of any finite dimension by generalizing Coles-Piani’s uncertainty relation and using majorization techniques. In particular, all of our results can be reduced to the case without the presence of quantum memory.

The close relationship between the information exclusion relation and the uncertainty principle has promoted mutual developments. In the applications of the uncertainty relation to the former, there have been usually two available methods: either through subtraction of the uncertainty relation in the presence of quantum memory or utilizing the concavity property of the entropy together with combinatorial techniques or certain symmetry. Our second goal in this work is to analyze these two methods and in particular, we will show that the second method together with a special combinatorial scheme enables us to find tighter bounds for the information exclusion principle. The underlined reason for effectiveness is due to the special composition of the mutual information. We will take full advantage of this phenomenon and apply a distinguished symmetry of cyclic permutations to derive new bounds, which would have been difficult to obtain without consideration of mutual information.

We also remark that the recent result^19^ for the sum of entropies is valid in the absence of quantum side information and cannot be extended to the cases with quantum memory by simply adding the conditional entropy between the measured particle and quantum memory. To resolve this difficulty, we use a different method in this paper to generalize the results of ref. 19 in Lemma 1 and Theorem 2 to allow for quantum memory.

## Results

We first consider the information exclusion principle for two observables, and then generalize it to multi-observable cases. After that we will show that our information exclusion relation gives a tighter bound, and the bound not only involves the *d* largest 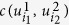 but contains all the overlaps 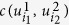 between bases of measurements.

We start with a qubit system to show our idea. The bound offered by Coles and Piani for two measurements does not improve the previous bounds for qubit systems. To see these, set 
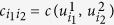
 for brevity, then the unitarity of overlaps between measurements implies that *c*_11_ + *c*_12_ = 1, *c*_11_ + *c*_21_ = 1, *c*_21_ + *c*_22_ = 1 and *c*_12_ + *c*_22_ = 1. Assuming *c*_11_ > *c*_12_, then *c*_11_ = *c*_22_ > *c*_12_ = *c*_21_, thus


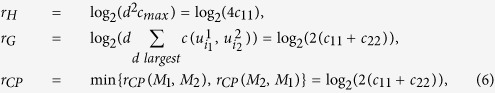


hence we get *r*_*H*_ = *r*_*G*_ = *r*_*CP*_ = log_2_(4*c*_11_) which says that the bounds of Hall, Grudka *et al*., and Coles and Piani coincide with each other in this case.

Our first result already strengthens the bound in this case. Recall the implicit bound from the tensor-product majorization relation^20^,^21^,^22^ is of the form


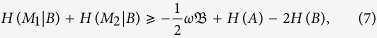


where the vectors 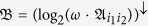 and 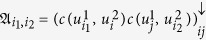 are of size *d*^2^. The symbol ↓ means re-arranging the components in descending order. The majorization vector bound *ω* for probability tensor distributions 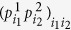 of state *ρ* is the *d*^2^-dimensional vector *ω* = (Ω_1_, Ω_1_ − Ω_2_, …, Ω_*d*_ − Ω_*d*−1_, 0, …, 0), where


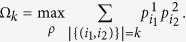


The bound means that


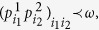


for any density matrix *ρ* and 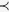 is defined by comparing the corresponding partial sums of the decreasingly rearranged vectors. Therefore *ω* only depends on 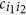
20. We remark that the quantity *H*(*A*) − 2*H*(*B*) assumes a similar role as that of *H*(*A*|*B*), which will be clarified in Theorem 2. As for more general case of *N* measurements, this quantity is replaced by (*N* − 1)*H*(*A*) − *NH*(*B*) in the place of *NH*(*A*|*B*). A proof of this relation will be given in the section of Methods. The following is our first improved information exclusion relation in a new form.

### Theorem 1

For any bipartite state *ρ*_*AB*_, let *M*_1_ and *M*_2_ be two measurements on system *A*, and *B* the quantum memory correlated to *A*, then


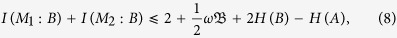


where *ω* is the majorization bound and 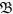 is defined in the paragraph under Eq. (7).

See Methods for a proof of Theorem 1.

Eq. (8) gives an implicit bound for the information exclusion relation, and it is tighter than log_2_(4*c*_*max*_) + 2*H*(*B*) − *H*(*A*) as our bound not only involves the maximal overlap between *M*_1_ and *M*_2_, but also the second largest element based on the construction of the universal uncertainty relation *ω*^21^,^22^. Majorization approach^21^,^22^ has been widely used in improving the lower bound of entropic uncertainty relation. The application in the information exclusion relation offers a new aspect of the majorization method. The new lower bound not only can be used for arbitrary nonnegative Schur-concave function^23^ such as Rényi entropy and Tsallis entropy^24^, but also provides insights to the relation among all the overlaps between measurements, which explains why it offers a better bound for both entropic uncertainty relations and information exclusion relations. We also remark that the new bound is still weaker than the one based on the optimal entropic uncertainty relation for qubits^25^.

**Figure 1:**
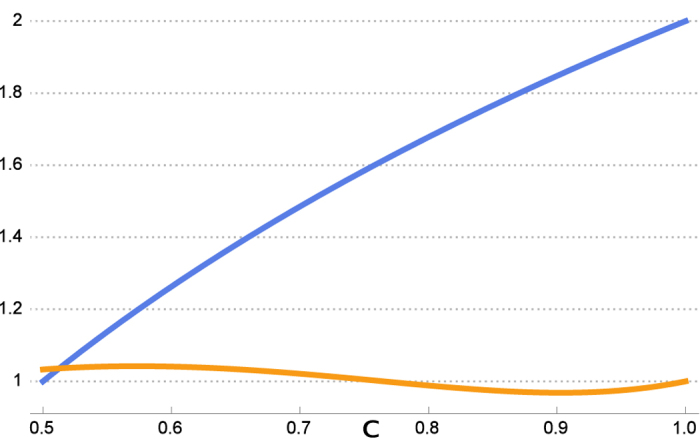
First comparison with Hall’s bound. The lower orange curve (our bound 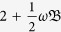) is tighter than the upper blue one (Hall’s bound *r*_*H*_) almost everywhere.

As an example, we consider the measurements *M*_1_ = {(1, 0), (0, 1)} and 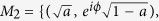 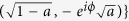. Our bound and log_2_ 4*c*_*max*_ for *ϕ* = *π*/2 with respect to *a* are shown in Fig. 1.

**Figure 2:**
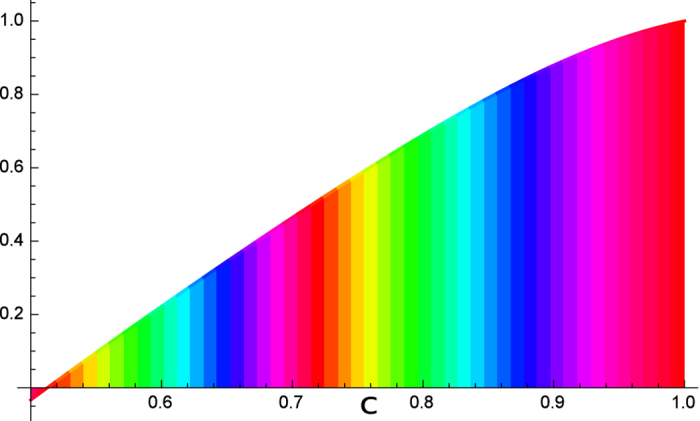
First comparison with Hall’s bound. The difference 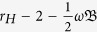 of our bound from Hall’s bound *r*_*H*_ for *a* ∈ [0.5, 1] is shown.

Figure 1 shows that our bound for qubit is better than the previous bounds *r*_*H*_ = *r*_*G*_ = *r*_*CP*_ almost everywhere. Using symmetry we only consider *a* in 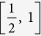. The common term 2*H*(*B*) − *H*(*A*) is omitted in the comparison. Further analysis of the bounds is given in Fig. 2.

Theorem 1 holds for any bipartite system and can be used for arbitrary two measurements *M*_*i*_ (*i* = 1, 2). For example, consider the qutrit state and a family of unitary matrices *U*(*θ*) = *M*(*θ*) *O*_3_*M*(*θ*)^† ^16,20 where


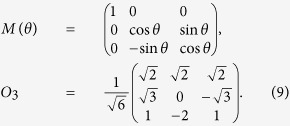


**Figure 3:**
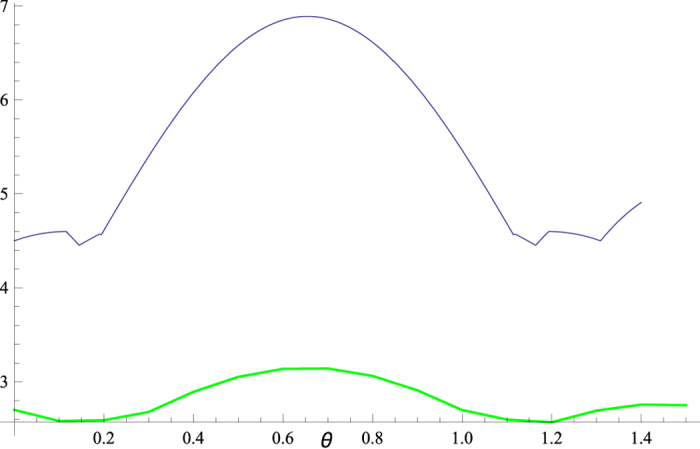
Comparison of our bound with that of Coles and Piani. Our bound 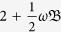 (lower in green) is better than Coles-Piani’s bound *r*_*CP*_ (upper in purple) everywhere.

Upon the same matrix *U*(*θ*), comparison between our bound 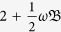 and Coles-Piani’s bound *r*_*CP*_ is depicted in Fig. 3.

In order to generalize the information exclusion relation to multi-measurements, we recall that the universal bound of tensor products of two probability distribution vectors can be computed by optimization over minors of the overlap matrix^21^,^22^. More generally for the multi-tensor product 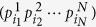 corresponding to measurement *M*_*m*_ on a fixed quantum state, there exists similarly a universal upper bound *ω*: 
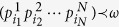
. Then we have the following lemma, which generalizes Eq. (7).

### Lemma 1

For any bipartite state *ρ*_*AB*_, let *M*_*m*_ (*m* = 1, 2, …, *N*) be *N* measurements on system *A*, and *B* the quantum memory correlated to *A*, then the following entropic uncertainty relation holds,


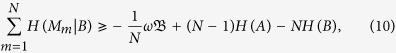


where *ω* is the *d*^*N*^-dimensional majorization bound for the *N* measurements *M*_*m*_ and 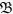 is the *d*^*N*^-dimensional vector 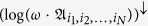 defined as follows. For each multi-index (*i*_1_, *i*_2_, …, *i*_*N*_), the *d*^*N*^-dimensional vector 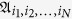 has entries of the form *c*(1, 2,…, *N*) *c*(2, 3, …, 1)…*c*(*N*, 1, …, *N* − 1) sorted in decreasing order with respect to the indices (*i*_1_, *i*_2_, …, *i*_*N*_) where 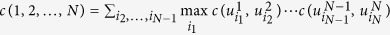.

See Methods for a proof of Lemma 1.

We remark that the *admixture bound* introduced in ref. 19 was based upon the majorization theory with the help of the action of the symmetric group, and it was shown that the bound outperforms previous results. However, the admixture bound cannot be extended to the entropic uncertainty relations in the presence of quantum memory for multiple measurements directly. Here we first use a new method to generalize the results of ref. 19 to allow for the quantum side information by mixing properties of the conditional entropy and Holevo inequality in Lemma 1. Moreover, by combining Lemma 1 with properties of the entropy we are able to give an enhanced information exclusion relation (see Theorem 2 for details).

The following theorem is obtained by subtracting the entropic uncertainty relation from the above result.

### Theorem 2

For any bipartite state *ρ*_*AB*_, let *M*_*m*_ (*m* = 1, 2, …, *N*) be *N* measurements on system *A*, and *B* the quantum memory correlated to *A*, then


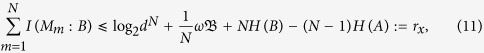


where 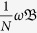 is defined in Eq. (10).

See Methods for a proof of Theorem 2.

Throughout this paper, we take *NH*(*B*) − (*N* − 1)*H*(*A*) instead of −(*N* − 1)*H*(*A*|*B*) as the variable that quantifies the amount of entanglement between measured particle and quantum memory since *NH*(*B*) − (*N* − 1)*H*(*A*) can outperform −(*N* − 1)*H*(*A*|*B*) numerically to some extent for entropic uncertainty relations.

Our new bound for multi-measurements offers an improvement than the bound recently given in ref. 18. Let us recall the information exclusion relation bound^18^ for multi-measurements (state-independent):


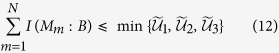


with the bounds 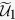, 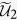 and 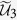 are defined as follows:


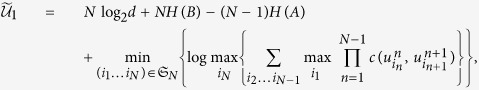



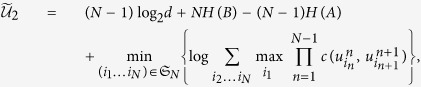



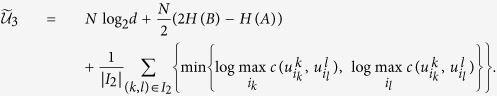


Here the first two maxima are taken over all permutations (*i*_1_ *i*_2_ … *i*_*N*_): *j* → *i*_*j*_, and the third is over all possible subsets 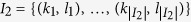 such that 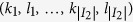 is a |*I*_2_|-permutation of 1, …, *N*. For example, (12), (23), …, (*N* − 1, *N*), (*N*1) are 2-permutation of 1, …, *N*. Clearly, 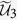 is the average value of all potential two-measurement combinations.

Using the permutation symmetry, we have the following Theorem which improves the bound 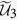.

### Theorem 3

Let *ρ*_*AB*_ be the bipartite density matrix with measurements *M*_*m*_ (*m* = 1, 2, …, *N*) on the system *A* with a quantum memory *B* as in Theorem 2, then


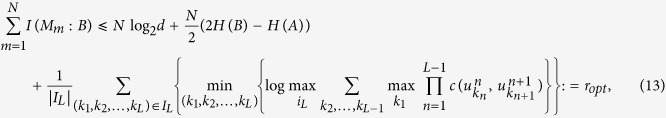


where the minimum is over all *L*-permutations of 1, …, *N* for *L* = 2, …, *N*.

In the above we have explained that the bound 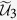 is obtained by taking the minimum over all possible 2-permutations of 1, 2, …, *N*, naturally our new bound *r*_*opt*_ in Theorem 3 is sharper than 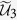 as we have considered all possible multi-permutations of 1, 2, …, *N*.

Now we compare 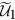 with *r*_*x*_. As an example in three-dimensional space, one chooses three measurements as follows^26^:


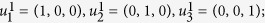



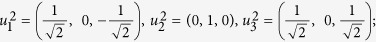



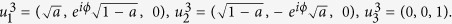


**Figure 4:**
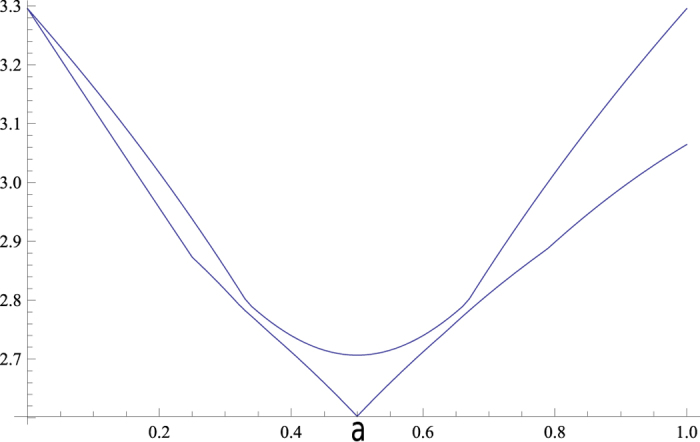
Comparison of our bound with that of Zhang *et al*. Our bound rx in the bottom is tighter than the top curve of Zhang’s bound 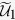.

Figure 4 shows the comparison when *a* changes and *ϕ* = *π*/2, where it is clear that *r*_*x*_ is better than 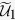.

**Figure 5:**
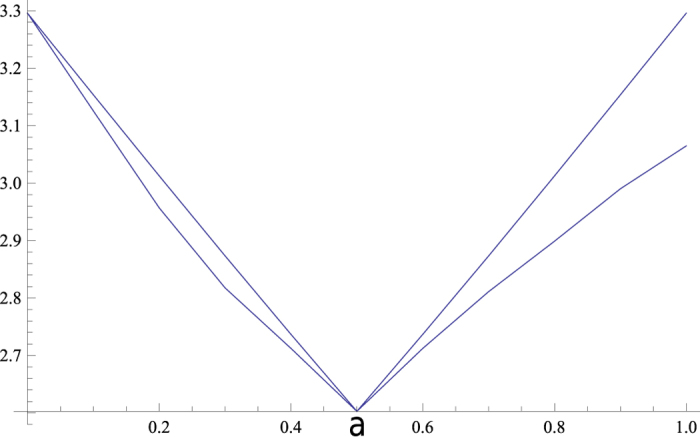
Comparison of our two bounds via combinatorial and majorization methods: the top curve is *r*_*opt*_ (combinatorial), while the lower curve is *r*_*x*_ (majorization).

The relationship between *r*_*opt*_ and *r*_*x*_ is sketched in Fig. 5. In this case *r*_*x*_ is better than *r*_*opt*_ for three measurements of dimension three, therefore min{*r*_*opt*_, *r*_*x*_} = min{*r*_*x*_}. Rigorous proof that *r*_*x*_ is always better than *r*_*opt*_ is nontrivial, since all the possible combinations of measurements less than *N* must be considered.

On the other hand, we can give a bound better than 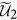. Recall that the concavity has been utilized in the formation of 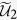, together with all possible combinations we will get following lemma (in order to simplify the process, we first consider three measurements, then generalize it to multiple measurements).

### Lemma 2

For any bipartite state *ρ*_*AB*_, let *M*_1_, *M*_2_, *M*_3_ be three measurements on system *A* in the presence of quantum memory *B*, then


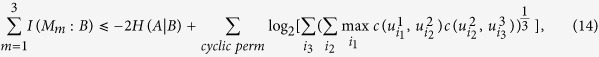


where the sum is over the three cyclic permutations of 1, 2, 3.

See Methods for a proof of Lemma 2.

Observe that the right hand side of Eq. (14) adds the sum of three terms


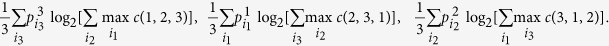


Naturally, we can also add 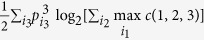 and 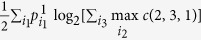. By the same method, consider all possible combination and denote the minimal as *r*_3*y*_. Similar for *N*-measurements, set the minimal bound under the concavity of logarithm function as *r*_*Ny*_, moreover let *r*_*y*_ = min_*m*_{*r*_*my*_} 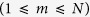, hence 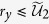, finally we get

### Theorem 4

For any bipartite state *ρ*_*AB*_, let *M*_*m*_ (*m* = 1, 2, …, *N*) be *N* measurements on system *A*, and let *B* be the quantum memory correlated to *A*, then


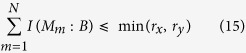


with 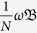 the same in Eq. (10). Since 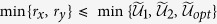 and all figures have shown our newly construct bound min {*r*_*x*_, *r*_*y*_} is tighter. Note that there is no clear relation between *r*_*x*_ and *r*_*y*_, while the bound *r*_*y*_ cannot be obtained by simply subtracting the bound of entropic uncertainty relations in the presence of quantum memory. Moreover, if *r*_*y*_ outperforms *r*_*x*_, then we can utilize *r*_*y*_ to achieve new bound for entropic uncertainty relations stronger than 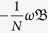.

## Conclusions

We have derived new bounds of the information exclusion relation for multi-measurements in the presence of quantum memory. The bounds are shown to be tighter than recently available bounds by detailed illustrations. Our bound is obtained by utilizing the concavity of the entropy function. The procedure has taken into account of all possible permutations of the measurements, thus offers a significant improvement than previous results which had only considered part of 2-permutations or combinations. Moreover, we have shown that majorization of the probability distributions for multi-measurements offers better bounds. In summary, we have formulated a systematic method of finding tighter bounds by combining the symmetry principle with majorization theory, all of which have been made easier in the context of mutual information. We remark that the new bounds can be easily computed by numerical computation.

## Methods

### Proof of Theorem 1

Recall that the quantum relative entropy 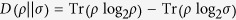 satisfies that 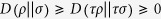 under any quantum channel *τ*. Denote by 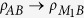 the quantum channel 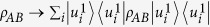, which is also 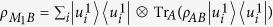. Note that both 
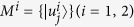
 are measurements on system *A*, we have that for a bipartite state *ρ*_*AB*_


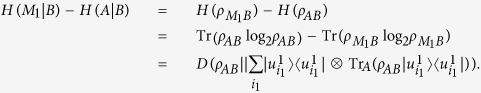


Note that 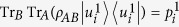, the probability distribution of the reduced state *ρ*_*A*_ under the measurement *M*_1_, so 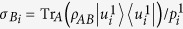 is a density matrix on the system *B*. Then the last expression can be written as


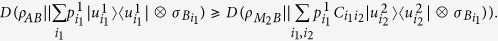


If system *B* is a classical register, then we can obtain


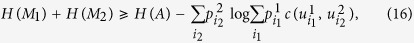


by swapping the indices *i*_1_ and *i*_2_, we get that


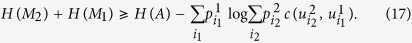


Their combination implies that


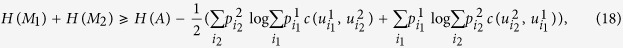


thus it follows from ref. 27 that


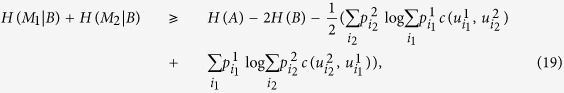


hence


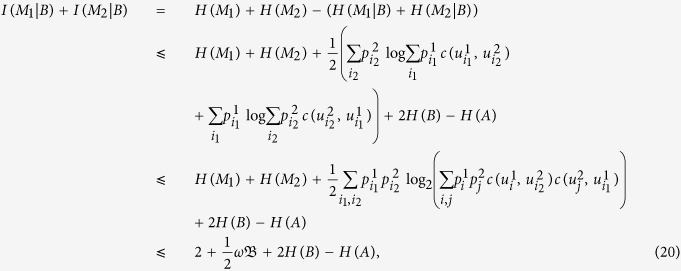


where the last inequality has used 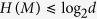 (*i* = 1, 2) and the vector 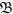 of length *d*^2^, whose entries 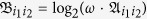 are arranged in decreasing order with respect to (*i*_1_, *i*_2_). Here the vector 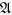 is defined by 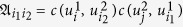 for each (*i*_1_, *i*_2_) and also sorted in decreasing order. Note that the extra term 2*H*(*B*) − *H*(*A*) is another quantity appearing on the right-hand side that describes the amount of entanglement between the measured particle and quantum memory besides −*H*(*A*|*B*).

We now derive the *information exclusion relation* for qubits in the form of 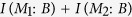 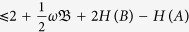, and this completes the proof.

### Proof of Lemma 1

Suppose we are given *N* measurements *M*_1_, …, *M*_*N*_ with orthonormal bases 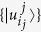. To simplify presentation we denote that


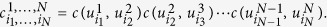


Then we have that^26^


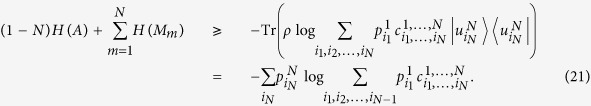


Then consider the action of the cyclic group of *N* permutations on indices 1, 2,..., *N*, and take the average can obtain the following inequality:


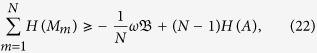


where the notations are the same as appeared in Eq. (10). Thus it follows from ref. 27 that


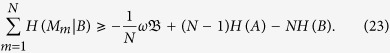


The proof is finished.

### Proof of Theorem 2

Similar to the proof of Theorem 1, due to *I*(*M*_*m*_ : *B*) = *H*(*M*_*m*_) − *H*(*M*_*m*_|*B*), thus we get


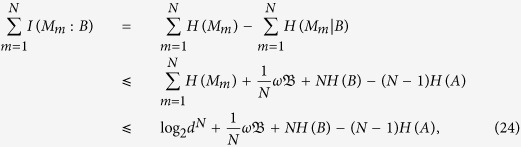


with the product 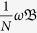 the same in Eq. (10).

### Proof of Lemma 2

First recall that for 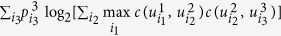 we have


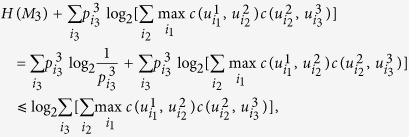


where we have used concavity of log. By the same method we then get


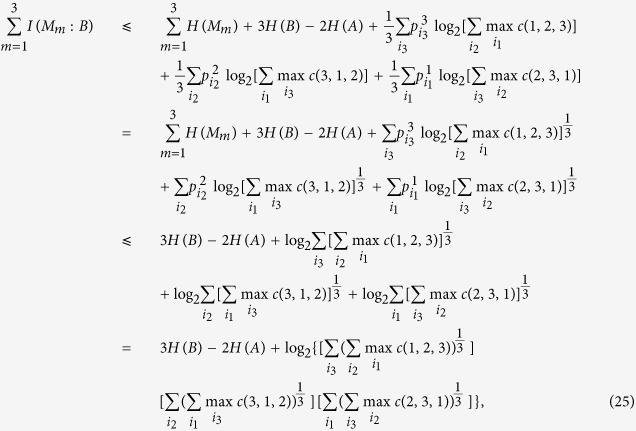


with *c*(1, 2, 3), *c*(2, 3, 1) and *c*(3, 1, 2) the same as in Eq. (14) and this completes the proof.

## Additional Information

**How to cite this article**: Xiao, Y. *et al*. Enhanced Information Exclusion Relations. *Sci. Rep.* **6**, 30440; doi: 10.1038/srep30440 (2016).
